# Development and validation of smartwatch-based activity recognition models for rigging crew workers on cable logging operations

**DOI:** 10.1371/journal.pone.0250624

**Published:** 2021-05-12

**Authors:** Eloise G. Zimbelman, Robert F. Keefe

**Affiliations:** Department of Forest, Rangeland and Fire Sciences, University of Idaho, Moscow, ID, United States of America; Fuzhou University, CHINA

## Abstract

Analysis of high-resolution inertial sensor and global navigation satellite system (GNSS) data collected by mobile and wearable devices is a relatively new methodology in forestry and safety research that provides opportunities for modeling work activities in greater detail than traditional time study analysis. The objective of this study was to evaluate whether smartwatch-based activity recognition models could quantify the activities of rigging crew workers setting and disconnecting log chokers on cable logging operations. Four productive cycle elements (*travel to log*, *set choker*, *travel away*, *clear*) were timed for choker setters and four productive cycle elements (*travel to log*, *unhook*, *travel away*, *clear*) were timed for chasers working at five logging sites in North Idaho. Each worker wore a smartwatch that recorded accelerometer data at 25 Hz. Random forest machine learning was used to develop predictive models that classified the different cycle elements based on features extracted from the smartwatch acceleration data using 15 sliding window sizes (1 to 15 s) and five window overlap levels (0%, 25%, 50%, 75%, and 90%). Models were compared using multiclass area under the Receiver Operating Characteristic (ROC) curve, or AUC. The best choker setter model was created using a 3-s window with 90% overlap and had sensitivity values ranging from 76.95% to 83.59% and precision values ranging from 41.42% to 97.08%. The best chaser model was created using a 1-s window with 90% overlap and had sensitivity values ranging from 71.95% to 82.75% and precision values ranging from 14.74% to 99.16%. These results have demonstrated the feasibility of quantifying forestry work activities using smartwatch-based activity recognition models, a basic step needed to develop real-time safety notifications associated with high-risk job functions and to advance subsequent, comparative analysis of health and safety metrics across stand, site, and work conditions.

## Introduction

Cable logging operations consist of felling, yarding, processing, and loading work phases [1], with the yarding phase often characterized as six distinct cycle elements (outhaul, lateral out, hookup, lateral in, inhaul, and unhook) [2]. Many of the yarding tasks, such as pulling the cable laterally as well as hooking and unhooking logs, are manual, which can cause physiological strain [3]. Logging workers are often fatally injured when struck by objects such as falling trees, limbs, or machines [4–10]. Contact with objects and equipment accounted for 70.9% of fatalities among logging workers in 2017 [9] and 82.1% of fatalities among logging workers in 2018 [10]. Hand fallers and choker setters are particularly susceptible to these “struck-by” incidents, which accounted for 51.3% of injuries among hand fallers and choker setters between July 2010 and June 2015 in Montana and Idaho [8]. In an analysis of cable logging accidents, Tsioras et al. [11] found that broken spar and anchor trees, bouncing cables, and falling objects contributed to the majority of accidents and most incidents occurred when workers were struck by or struck against an object. While the increased mechanization of logging has generally led to a decrease in injury rates, ground crew members working alongside machines, rigging crew workers, and hand fallers are still at risk [4, 6, 8, 12] and may benefit from the use of a variety of positioning and wearable sensor-based technologies that increase situational awareness and reduce accidents.

Monitoring the current activities, safety status and location of individuals relative to workplace hazards on logging operations could be accomplished through (1) real-time location-sharing methods based on GNSS-RF (global navigation satellite system (GNSS) positioning paired with radio frequency (RF) transmission) for use in remote areas [13–17], (2) activity recognition modeling and incident detection, or (3) a combination of both. Human activity recognition involves using wearable sensors to distinguish between human physical activities. Most activity recognition models have been developed for everyday activities, such as walking, sitting, lying, standing, and other common physical movements referred to as activities of daily living (ADLs) [18–21], as well as for recreation and fitness applications [22–24]. Many smartphones and smartwatches are equipped with a variety of embedded sensors such as GNSS chips, accelerometers, gyroscopes, barometers, magnetometers, thermometers, decibel meters (microphones), and optical heart rate sensors [25–30]. Although a variety of purpose-built sensors have been developed, smartphones [26, 28, 31] and smartwatches [27, 29, 32–35] are popular for activity recognition modeling because they are ubiquitous and unobtrusive. Leveraging a variety of wearable and positioning sensors to develop occupational activity recognition models in forestry is a first step toward active monitoring that utilizes subsequent model predictions to help inform algorithms identifying falls or high-risk activities. Real-time prediction of work cycle elements represents an initial step toward informing smart, location- and activity-aware algorithms and alerts associated with detecting incidents and periods of elevated health and safety concern.

**Fig 1:**
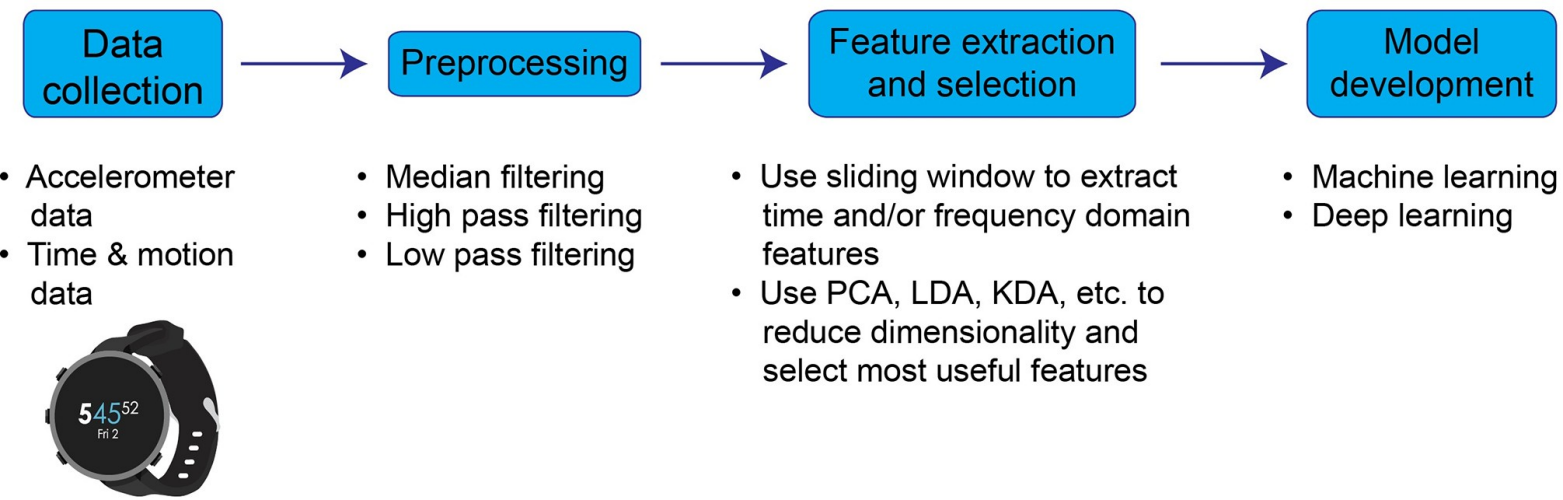
Outline of the general activity recognition model development process. Steps involved typically include (1) collecting time study data to pair with wearable sensor measurements, (2) preprocessing the data through filtering, (3) extracting time and/or frequency domain features using a sliding window and then selecting relevant features with which to build models, and (4) developing activity recognition models using machine learning or deep learning techniques. Ultimately, models may be programmed into apps on smartphones and smartwatches and subsequently used to characterize work activities in real-time to inform health and safety notifications.

Development of activity recognition models generally consists of data collection, preprocessing, feature extraction and selection, and model development (Fig 1) [28, 36, 37]. Due to the advent of microelectromechanical systems (MEMS), inertial sensors have become smaller, more accurate, and less expensive and have been integrated into a variety of wearable sensors [38]. Data from these sensors is collected while users perform the activities of interest and is typically annotated with observed start and stop times. Preprocessing commonly consists of median filtering to remove noise spikes [39] and low pass or high pass filtering to isolate acceleration due to gravity from body acceleration [39–41]. To extract features for model development, a moving, or sliding, window is advanced through the dataset, defining subsets of the data from which relevant time (e.g., mean, median, variance, standard deviation, range, skewness and kurtosis) or frequency (e.g., Fast Fourier Transform and Discrete Transform coefficients) domain features are calculated [30, 31, 42, 43]. In order to reduce dimensionality and select the most useful features, a variety of techniques such as principal component analysis (PCA), singular value decomposition (SVD), linear discriminant analysis (LDA), or kernel discriminant analysis (KDA) can be used [41, 44, 45]. Finally, the extracted features are used to develop activity recognition models, often using machine learning algorithms such as Decision Trees, Random Forests (RFs), Support Vector Machines (SVMs), k-Nearest Neighbors (k-NN), Naïve Bayes, k-means, Hidden Markov Models (HMMs), Gaussian Mixture Models (GMMs), artificial neural networks (ANNs), and multilayer perceptron (MLP) [28, 31, 43, 46]. More recently, deep learning methods such as Restricted Boltzmann Machine, Autoencoders, Convolutional Neural Networks, and Recurrent Neural Networks have been shown to improve human activity recognition model performance compared to classical machine learning algorithms [47–50]. While deep learning can overcome some of the drawbacks of traditional machine learning by automatically extracting features and using more complex features [48–50], they are generally more computationally expensive and thus have not been widely implemented on resource-limited devices such as smartwatches and smartphones [47].

Model accuracy is thus affected by a variety of factors, such as the type and quality of sensors in the devices, sampling rate, device location on the body, machine learning algorithms employed for model development, features used for classification, and sliding window size used to extract these features [31, 42, 51]. In terms of window size, there is a tradeoff between detection times and recognition performance since smaller windows allow faster recognition speed [51] but longer windows have been shown to improve recognition performance for more complex, less repetitive activities [34]. In evaluating this tradeoff, Banos et al. [51] compared activity recognition models created with windows ranging in size from 0.25 s to 7 s and found that windows of 1–2 s provided the best accuracy while allowing for quick detection times. The amount of overlap between successive sliding windows also affects model accuracy, with larger overlap often leading to better model performance but at the cost of increased computational load [45]. Models with 50% overlap are common [20, 40, 52–55], but high accuracies can be obtained using nonoverlapping windows [29, 34, 51, 56]. Recently, “online” activity recognition, which refers to implementing the entire classification process (i.e., data collection and pre-processing, feature extraction, and classification) locally on the device, has been investigated to attain near real-time classifications [31, 42]. Online recognition systems have been shown to have classification accuracies of 45.4–98.1% [42, 57, 58].

While most activity recognition models are specific to ADLs, the high-resolution data collected by these devices may also be useful for quantifying work activities. A limited number of studies have developed activity recognition for occupational activities based on wearable sensors, primarily for construction [59–63], while others have used visual observation or vision sensors to detect activities in the workplace [64, 65]. In natural resources, this approach has recently been proposed by Keefe et al. [30] and Pierzchała et al. [66] developed a method of automatically distinguishing between work phases in cable yarding by fusing data from multiple sensors. New GNSS-enabled smartwatches may offer lightweight alternatives to smartphone-based activity recognition models and may provide additional data that could supplement and improve these models. These types of wearable-based predictive models that quantify work activities on logging operations could inform loggers and equipment operators about their own or their coworkers’ job activity status in near real-time, helping to increase situational awareness and safety on active timber sales. New, inexpensive mesh network communications technologies, such as those from goTenna Inc (Brooklyn, NY, USA), enable location- and data-sharing by connecting to smartphones via Bluetooth and allowing users to communicate through radio frequency. In addition to facilitating off-the-grid location-sharing, these devices may also be useful for sharing worker safety status derived from activity recognition models. The record of high-resolution data that results from activity recognition may also form the basis for quantifying occupational health and safety conditions in comparative analyses that span forest stand, site, and work conditions.

Time and motion studies [67] have been used extensively in conventional forest operations research to quantify productive work cycle elements and delay [2, 68–73]. By defining and analyzing the individual work cycle elements performed by equipment or by individuals engaged in motor-manual operations, an objective of time study research is often to identify opportunities for improving occupational production rates and reducing delay time [1, 2, 71, 72, 74]. Time study analyses are used as the basis for regression [1, 2, 69, 71, 74, 75] and machine learning [76, 77] models that predict work cycle time as a function of stand or site conditions. In order to estimate logging costs per unit wood volume, machine rate estimates determined using methods outlined by Miyata [78] and Brinker et al. [79] are paired with these cycle time prediction models [1, 71, 74, 75, 80]. In recent years, GNSS has been used in time studies to automate the estimation of cycle times [81], calculate machine productivity [82], characterize machine movements [83], and improve operational monitoring [84]. Additionally, time and motion studies have been conducted using both GNSS receivers and accelerometers to monitor tree planting [85], characterize manual felling using brush cutters [86], distinguish between chipping tasks [87], and monitor tilt and motion of various harvesters and forwarders in order to analyze operating conditions [88]. The availability of high-resolution sensor data collected seamlessly from GNSS-enabled mobile and wearable devices in real-time provides an opportunity to further model forestry work activities in greater detail than has been done using traditional methods, while simultaneously providing the basis for improved characterization of digital health and safety.

Prior research evaluating use of wearable sensors to monitor and model forestry work activities includes a small body of recent literature. Fitness and sleep bands have been used (1) to monitor the physical activity and sleep patterns of forestry workers in order to understand how these factors may contribute to workplace hazards [89], and (2) to predict forestry worker fatigue by comparing heart rate and step count data to reaction and decision-making times [90]. Smartwatches paired with heart rate monitor chest straps have been used to evaluate workload associated with manual tree felling [91], while external accelerometers attached to machines have been used to develop ANNs that classify the activities of manually-driven bandsaws [92] and recognize activities associated with manual felling [93]. Preliminary activity recognition models have been developed for cable yarding work phases using a combination of smartphone sensor (global positioning system (GPS) and inertial measurement unit (IMU)) and camera data [66]. However, activity recognition models have yet to be developed for other forestry work positions, such as rigging crew workers setting and disconnecting log chokers. Furthermore, smartwatches have not previously been used in forestry activity recognition, so it is unknown how the prediction accuracy of models developed using these devices will compare to smartphone-based models. Specifically, smartwatches may record different movement patterns than smartphones due in part to different device locations on the body (i.e., wrist vs. hip) [27, 34]. In this study, we developed smartwatch-based activity recognition models for rigging crew workers on cable logging operations in order to address two specific research objectives. Our first objective was to develop models that predict choker setter rigging crew work activities with at least 80% sensitivity. Our second objective was to develop models that predict chaser rigging crew work activities with at least 80% sensitivity. Random forest machine learning was used to develop watch-based activity recognition models based on time and motion study data collected on five active timber sales in North Idaho, USA. Model accuracy was calculated based on the percent of time work elements were predicted correctly.

## Materials and methods

### Ethics statement

Fourteen loggers voluntarily participated in this study. Prior to data collection, the experimental protocol was approved by the University of Idaho Institutional Review Board (IRB number: 18–202). Participants received both oral and written information regarding the study design and provided their written, informed consent. Participants were selected based on available, operational cable-logging activities occurring in the North Idaho region during the sampling period and reflect the general demographics of the study population. While we did not collect demographic information from our participants, recent sampling in the region has shown that 55.4% of the logging workforce in Idaho is 50 or more years of age [17]. However, rigging crew workers are generally younger than the median age.

### Data collection and processing

Time and motion study (i.e., observational elemental time analysis) data was collected in conjunction with GNSS watch sensor data using two days of sampling on each of five timber sales. Timber sales occurred on state and industrial cable logging operations. Choker setters (who are responsible for setting chokers on logs to be yarded) and chasers (who are responsible for disconnecting chokers from yarded logs) were observed visually. Four productive cycle elements (*travel to log*, *set choker*, *travel away*, *clear*) were timed for the choker setters and four productive cycle elements (*travel to log*, *unhook*, *travel away*, *clear*) were timed for the chasers (Table 1). For the choker setter activities, *travel to log* began when the choker setter started walking toward the carriage to grab the chokers. *Set choker* began when the choker setter arrived at a log and began preparing chokers. *Travel away* began when the choker setter finished setting chokers and started to walk away from the log. *Clear* began when the choker setter stopped walking away from the log and was “in the clear”. For the chaser activities, *travel to log* began when the chaser started walking toward the landed logs. *Unhook* began when the chaser reached for the chokers to begin unhooking them. *Travel away* began when the chaser finished unhooking the chokers and started to walk away from the log(s). *Clear* began when the chaser stopped walking away from the logs and was “in the clear”. For both the choker setter and chaser, *clear* included everything workers did outside of the other three work elements. In order to develop a generalized model for functional use, minor delay events were included within the relevant work element during which they occurred. The clock on a Google Pixel smartphone was used to record the true start and stop times for each work activity cycle using the TimeStamp application (version 0.4.0) [94]. Workers wore Garmin Fenix 5S Plus watches, which record GNSS locations, heart rate, and raw accelerometer data, on their non-dominant wrist. All sensor data was recorded on the Garmin watches using the RawLogger application (version 1.0.20190520a) [95] from Garmin Connect. The accelerometer sensor data was collected at a 25-Hz frequency, while other sensors were recorded at a 1-Hz frequency, which are the default frequencies within the RawLogger application. All sensor data was exported as a Garmin FIT file and subsequently converted into a *.*csv* file. Only the watch accelerometer data, collected at 25 Hz and recorded in thousandths of a gravity (mgn), was used in model development.

**Table 1:** Summary of productive cycle elements for choker setter and chaser work activities.

Position	Activity	Activity begins when subject:
Choker setter	Travel to log	Initiates walking toward carriage to acquire chokers
	Set choker	Arrives at log
	Travel away	Finishes setting choker
	Clear	Stops walking away when safely “in the clear”
Chaser	Travel to log	Initiates walking toward the landed logs
	Unhook	Reaches for the chokers to begin unhooking
	Travel away	Finishes unhooking chokers
	Clear	Stops walking away when safely “in the clear”

All data processing, analysis and model development was done in the R statistical programming environment, version 4.0.0 [96]. After data collection, all observations in the datasets were labeled according to the manually recorded start and stop times. Specifically, each observation whose timestamp fell within the start and stop time for a particular activity cycle was assigned a label for that activity element (i.e., *travel to log*, *set choker*, *travel away*, *clear*, etc.). Delay times were included as the corresponding productive work element because the majority of delays fell within *clear*, included a diverse range of physical movements associated with the workers, and because of the intended final use of a general model in continuous, real-time prediction. After labeling, the raw acceleration values (in the x, y, and z dimensions) were filtered using a Finite Impulse Response (FIR) bandpass filter of order 8. Filter band edges were 0.5 and 0.9. Rather than using the x, y, and z values of the acceleration sensor, the acceleration magnitude was calculated using Eq (1) and used in an effort to reduce the effects of orientation on recognition performance:

$$A_{mag}=\sqrt{A_{x}^{2}+A_{y}^{2}+A_{z}^{2}}$$ (1)

Where *A*_*mag*_ is the filtered overall acceleration magnitude, and *A*_*x*_, *A*_*y*_, and *A*_*z*_ are the filtered acceleration sensor values in the x, y, and z dimensions, respectively.

### Activity recognition model development

**Fig 2:**
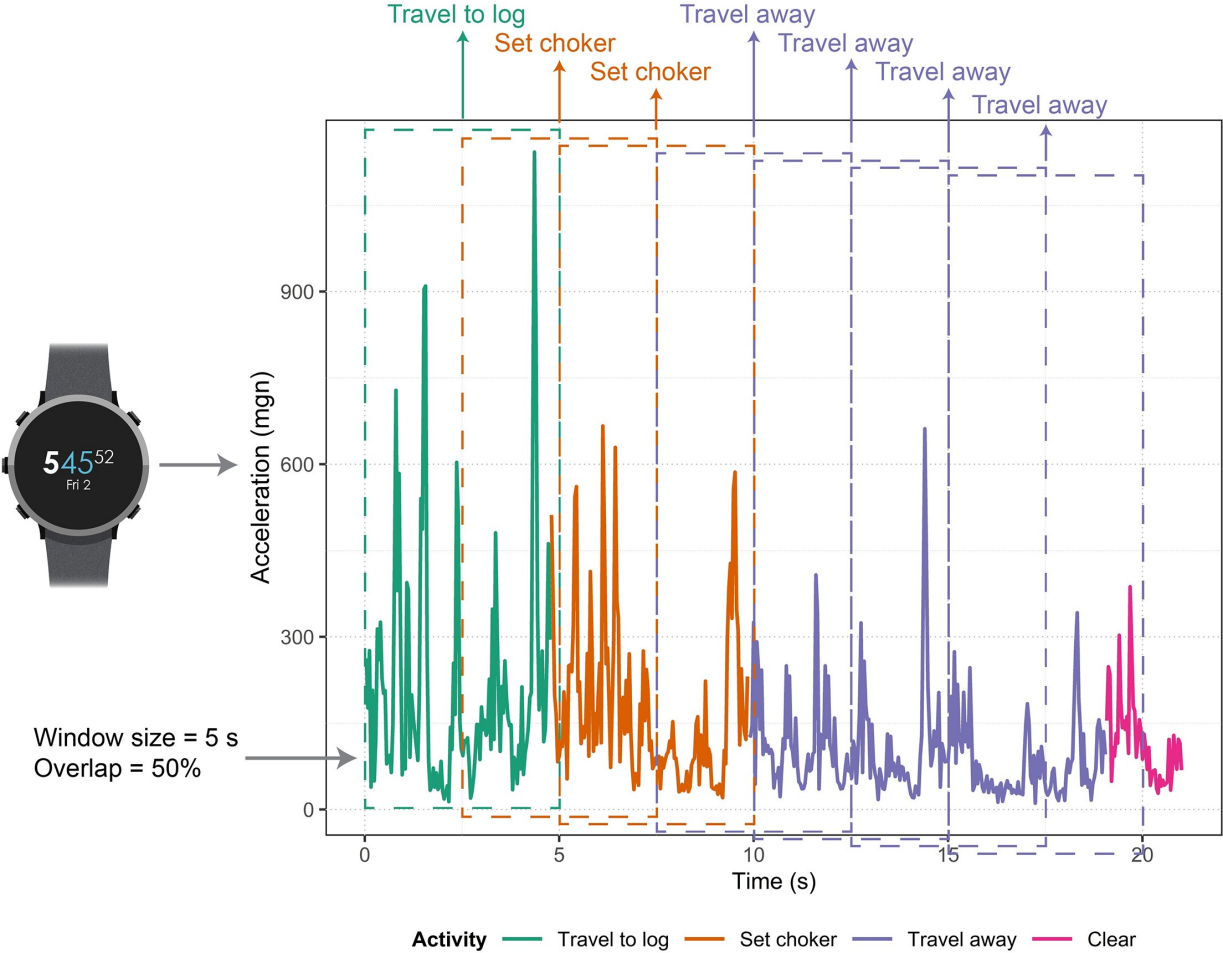
Overview of a hypothetical choker setter activity recognition model running on a smartwatch. The activity recognition model depicted is using a 5-s window with 50% overlap to predict the four work activities. The figure shows filtered acceleration magnitude data, which is colored according to the actual work cycles. Each time a window (shown as rectangles with dashed lines) is used to extract features, the model predicts the work cycle (shown as labels above the windows).

Ten time domain features (mean, standard deviation, maximum, minimum, median absolute deviation, mean absolute deviation, skewness, interquartile range, range, and kurtosis) were extracted from the filtered acceleration magnitude values from both the choker setter and chaser work activity data using 15 different sizes of sliding windows (ranging from 1 to 15 s). For example, using a 3-s window and data recorded at 25 Hz, features were calculated using the previous 75 observations (representing 3 s of data) each time the window was advanced. Windows with 0%, 25%, 50%, 75%, and 90% overlap were used, resulting in five feature extraction methods for each window size. For instance, using 25% overlap meant that the next window did not begin until the current window was 75% complete. After filtering and applying sliding windows, the resulting datasets were separated into 2/3 training and 1/3 testing data. Data was separated randomly, but the relative ratios of each activity were preserved because the data was highly imbalanced. The randomForest function in the R randomForest package (version 4.6–14) [97] was used to create random forest models to predict the four work cycle elements of both the choker setter and chaser based on the sensor measurements (Fig 2). Because the data was imbalanced, models were created using stratified sampling according to activity, with sample size based on the number of instances of the least common activity. In terms of the choker setter models, the least common activity was *travel away*. The least common chaser activity was *travel to log*. Random forest models can be tuned via a variety of parameters, such as the number of trees to grow (*n*_*tree*_) and the number of predictor variables randomly selected at each node (*m*_*try*_) [97, 98]. In this study, random forest models were created using 150 trees, since previous work has suggested that using 64–128 trees is appropriate for balancing performance and processing time [99]. Because varying *m*_*try*_ generally does not have a significant effect on model performance [97, 100], models in this study were built with the default value of *m*_*try*_ (the square root of the total number of variables). The relationship between the number of trees and model accuracy was evaluated using the out-of-bag (OOB) sample error rates calculated internally by the random forest algorithm.

Initially, choker setter and chaser models were created for the 15 window sizes using 90% overlap and all ten features, but model accuracy was poor. Thus, principal component analysis (PCA) was used to reduce the number of features in the models. The same ten time domain features described above were calculated for each activity cycle for both the choker setter and chaser datasets using the filtered acceleration magnitude values. PCA was performed on these ten features for both the choker setter and chaser datasets. Principal components (PCs) that accounted for > 95% of the variation in each dataset were considered and individual variables with loadings > |0.4| within these PCs were used as predictors in the final models.

After selecting model predictors, a total of 75 choker setter models and 75 chaser models were created using the 15 window sizes for each of the five levels of overlap. Models were created using the training datasets and the confusionMatrix function in the R caret package (version 6.0–85) [101] was used to calculate a variety of model accuracy metrics based on the testing datasets. All models were initially evaluated using sensitivity, specificity, and precision to compare the effects of overlap levels and window sizes. Sensitivity, specificity, and precision were calculated using Eqs (2–4):

$$Se=\frac{TP}{TP+FN}$$ (2)

$$Sp=\frac{TN}{TN+FP}$$ (3)

$$Pr=\frac{TP}{TP+FP}$$ (4)

Where *Se* is the sensitivity, *Sp* is the specificity, and *Pr* is the precision. *TP* is the number of true positives (i.e., the number of correctly classified instances of a given class), *TN* is the number of true negatives (i.e., for a given class, the number of instances of all other classes that are classified as anything other than the class of interest), *FP* is the number of false positives (i.e., the number of instances that are incorrectly classified as belonging to a given class), and *FN* is the number of false negatives (i.e., the number of instances of a given class that are incorrectly classified as a different class). Sensitivity, specificity, and precision were then converted to and reported as percentages. Sensitivity, or recall, is the true positive rate and represents the percentage of correctly identified activities of a particular class [102]. Specificity is the true negative rate and measures the percentage of correctly detected negative occurrences of a particular class [102]. Precision, or positive predictive value, measures the percentage of detected instances of an activity that represents a real occurrence [102]. Finally, the multiclass area under the Receiver Operating Characteristic (ROC) curve, or AUC, was calculated for each model. The AUC corresponds to the probability that a classifier will rank a randomly chosen positive instance higher than a randomly chosen negative instance, with higher AUC values indicating better performance [103]. AUC is a common criterion for evaluating the performance of classification algorithms [104, 105]. It has also been shown to be relatively robust to data imbalance [106–108]. The multiclass.roc function in the R pROC package (version 1.16.2) [109] was used to compute the multiclass AUC according to the method defined by Hand and Till [110]. The multiclass AUC value was then used to compare models and choose the best window size and overlap level for the choker setter and chaser models. Final models were evaluated based on the three metrics described previously (sensitivity, specificity, and precision) as well as F_1_ values and balanced accuracy. F_1_ values and balanced accuracy were calculated using Eqs (5 and 6):

$$F_{1}=\frac{2*Pr*Se}{Pr+Se}$$ (5)

$$BA=\frac{Se+Sp}{2}$$ (6)

Where *F*_*1*_ is the F_1_ value and *BA* is the balanced accuracy. *Pr* is the precision, *Se* is the sensitivity, and *Sp* is the specificity. The F_1_ value represents the harmonic mean of precision and recall (sensitivity) and is generally thought to be more robust when dealing with imbalanced classes [51]. It ranges from zero to one, with zero representing no capacity for recognition and one corresponding to perfect recognition [51]. The F_1_ value was calculated using the rate of precision and sensitivity (rather than the percent). Balanced accuracy is simply the mean of sensitivity and specificity and was calculated using the percentage values of these two metrics.

## Results

### Work activity cycle times

For the choker setter work activities, *travel to log* averaged 17.27 s (s = 12.30 s), *set choker* averaged 19.80 s (s = 16.06 s), *travel away* averaged 13.11 s (s = 6.83 s), and *clear* averaged 220.31 s (s = 519.17 s) (Table 2). For the chaser work activities, *travel to log* averaged 4.59 s (s = 4.42 s), *unhook* averaged 8.00 s (s = 6.42 s), *travel away* averaged 6.69 s (s = 3.73 s), and *clear* averaged 264.48 s (s = 481.49 s) (Table 2). Mean work activity cycle times for the chaser were generally shorter than the mean cycle times for the choker setter, and the maximum cycle times tended to be slightly longer for the choker setter activities compared to the chaser activities (Table 2). When expressed as a percentage of the mean elemental time for individual work cycles other than *clear*, the coefficient of variation (CV) ranged from 52.11% to 81.09% for the choker setter and from 55.70% to 96.28% for the chaser (Table 2). *Clear* was the only element for which the CV > 100% for both the choker setter and chaser (Table 2).

**Table 2:** Summary statistics (in seconds) of cycle times for choker setter and chaser work activities.

Position	Activity	Mean (s)	SD (s)	CV (%)	Range (s)	Median (s)	1st Quartile (s)	3rd Quartile (s)
Choker setter	Travel to log	17.27	12.30	71.23	0.72–105.56	13.96	9.06	21.82
	Set choker	19.80	16.06	81.09	1.14–188.21	14.73	9.46	24.61
	Travel away	13.11	6.83	52.11	2.5–55.74	11.87	8.74	15.44
	Clear	220.31	519.17	235.65	5.7–8441.34	120.22	93.55	166.14
Chaser	Travel to log	4.59	4.42	96.28	0.54–89.92	3.85	2.74	5.49
	Unhook	8.00	6.42	80.31	0.86–71.58	6.52	4.17	10.07
	Travel away	6.69	3.73	55.70	1.19–26.14	5.61	3.95	8.85
	Clear	264.48	481.49	182.06	4.93–8238.2	174.55	144.49	229.41

### Participant data

Due to the observational nature of data collection, the amount of data used to train and test the random forest models varied between participants. The average amount of data per choker setter ranged from 0.80 hrs (s = 0.01 hrs) to 6.74 hrs (s = 0.05 hrs) for training and from 0.40 hrs (s = 0.01 hrs) to 3.31 hrs (s = 0.05 hrs) for testing. The average amount of data per chaser ranged from 0.80 hrs (s = 0.02 hrs) to 4.59 hrs (s = 0.04 hrs) for training and from 0.39 hrs (s = 0.02 hrs) to 2.26 hrs (s = 0.04 hrs) for testing. When expressed as a percentage of the mean training and testing sample times for individual choker setters, the standard deviation ranged from 0.69% to 1.65% of the training sample times and from 1.39% to 3.34% of the testing sample times. Similarly, for individual chasers, the standard deviation ranged from 0.93% to 2.04% of the training sample times and from 1.90% to 4.10% of the testing sample times.

### Principal component analysis

**Fig 3:**
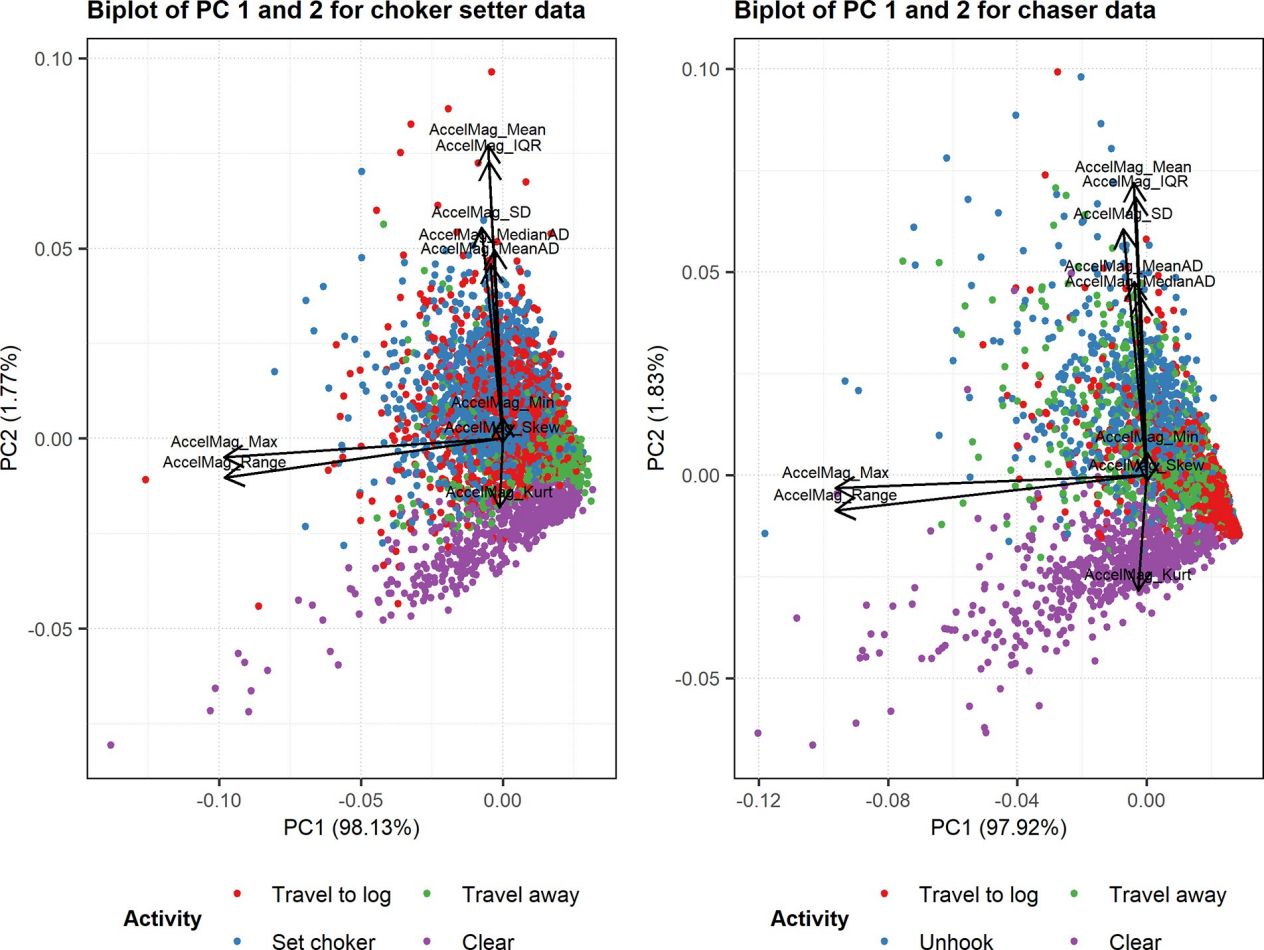
Biplots of PCs 1 and 2 for the choker setter and chaser datasets. The color of points on each plot indicates work cycle element categories.

In terms of the choker setter, the first PC accounted for 98.13% of the variation in the dataset and the only variables with loadings > |0.4| in the first PC were the acceleration maximum and range (Table 3). Similarly, in terms of the chaser, the first PC accounted for 97.92% of the variation in the dataset and the only variables with loadings > |0.4| in the first PC were the acceleration maximum and range (Table 3). Thus, acceleration maximum and range were selected as predictors in the final models for both workers. Biplots of the first two PCs for both the choker setter and chaser datasets illustrate the strong effect of acceleration maximum and range on the two datasets (Fig 3).

**Table 3:** Summary of choker setter and chaser PCA results.

	Position	Choker setter				Chaser			
	PC	PC1	PC2	PC3	PC4	PC1	PC2	PC3	PC4
	Percent of Variance	*98*.*13%*	*1*.*77%*	*0*.*05%*	*0*.*03%*	*97*.*92%*	*1*.*83%*	*0*.*14%*	*0*.*08%*
**Variable**	Mean	-0.038	**0.554**	0.235	-0.303	-0.030	**0.527**	-0.221	0.029
	Standard deviation	-0.054	0.398	**-0.686**	-0.353	-0.053	**0.443**	0.395	**-0.624**
	Maximum	**-0.705**	-0.036	0.056	-0.062	**-0.705**	-0.024	-0.014	0.028
	Minimum	0.000	0.039	0.092	-0.158	0.000	0.041	-0.042	-0.002
	Median absolute deviation	-0.021	0.356	0.354	0.163	-0.014	0.321	-0.299	0.285
	Mean absolute deviation	-0.031	0.329	-0.264	-0.063	-0.028	0.349	0.125	-0.278
	Skewness	-0.001	-0.009	0.010	-0.036	-0.001	-0.011	-0.020	-0.022
	Interquartile range	-0.035	**0.524**	0.296	0.345	-0.025	**0.501**	-0.323	0.284
	Kurtosis	-0.009	-0.131	**0.422**	**-0.771**	-0.020	-0.208	**-0.763**	**-0.607**
	Range	**-0.705**	-0.074	-0.036	0.096	**-0.705**	-0.064	0.028	0.030

The percentages of variance for the first four PCs as well as the individual loadings for each variable for the first four PCs are shown. Numbers in **bold** indicate variables with loadings > |0.4|.

### Number of trees

**Fig 4:**
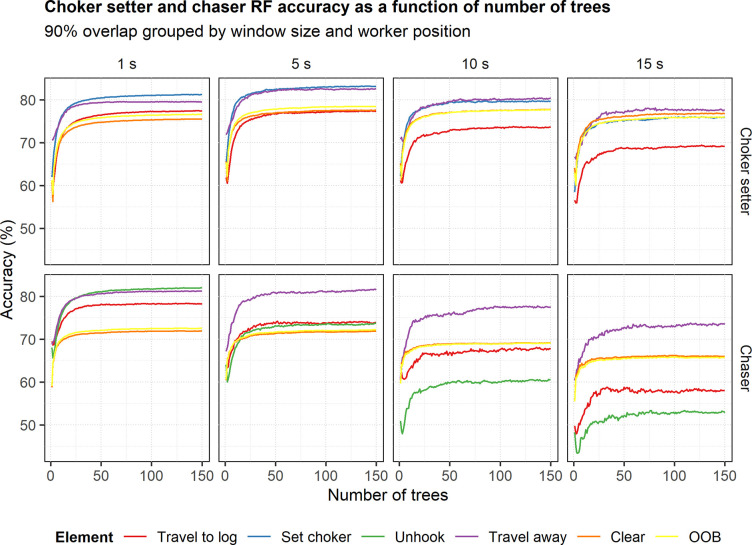
Choker setter and chaser random forest model accuracy as a function of the number of trees. The plots are grouped by worker type (choker setter or chaser) and window size. Line color indicates overall model (OOB) accuracy as well as accuracy for the work cycle elements. Only the 90% overlap of the 1-, 5-, 10- and 15-s windows are shown.

At the 90% overlap level, the classification accuracies determined internally by the random forest algorithm for both the choker setter and chaser models leveled off after 25–50 trees for most window sizes (Fig 4), and similar trends were observed for the other overlap levels (not shown). This suggests that building our models with 150 trees was sufficient.

### Choker setter models

**Fig 5:**
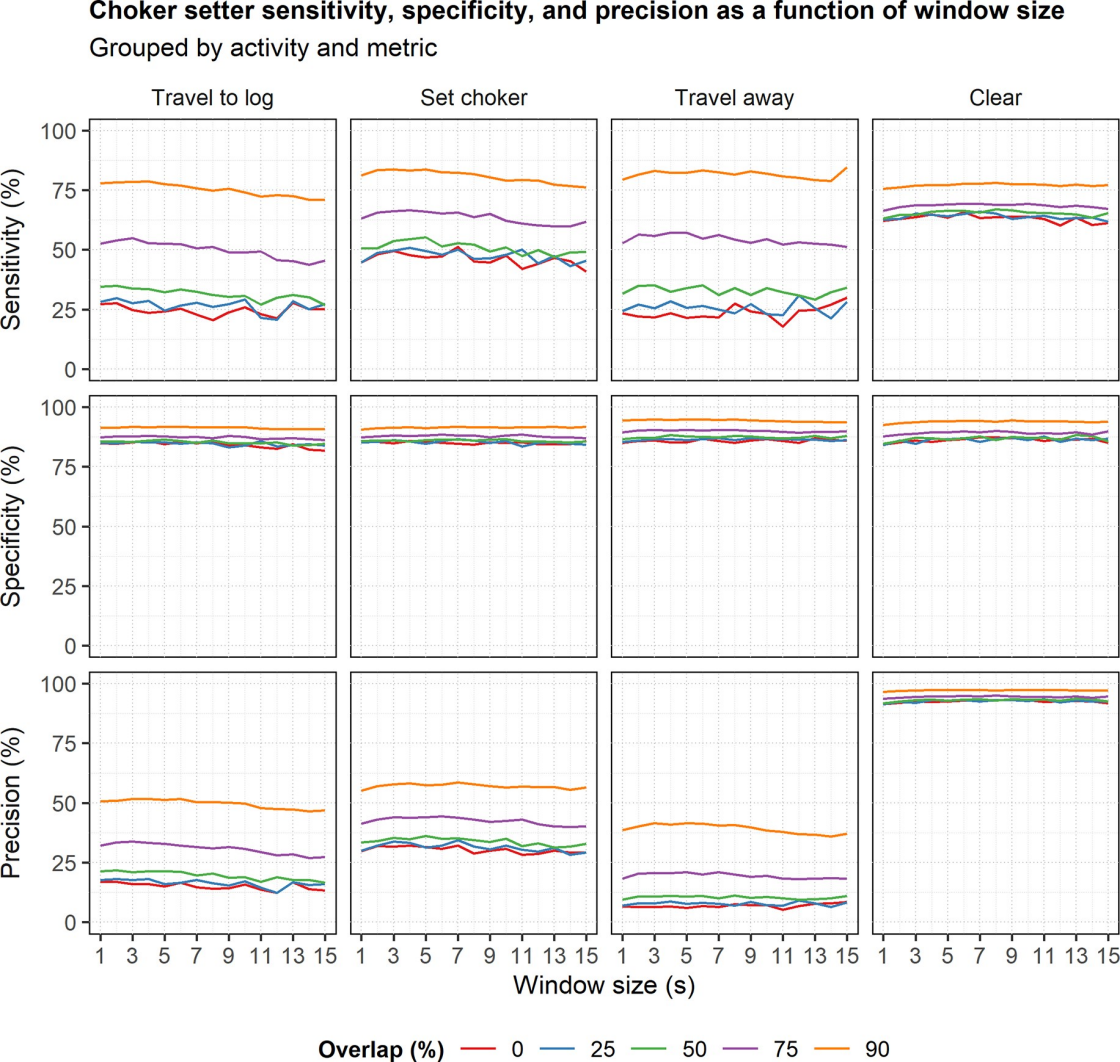
Choker setter sensitivity, specificity, and precision as a function of window size. The plots are grouped by metric and work activity. Line color indicates window overlap level.

The sensitivity, specificity, and precision of the choker setter models were highest when using sliding windows with 90% overlap for all activities and window sizes (Fig 5). Using sliding windows with 75% overlap resulted in the second-highest values of these same metrics for all activities and window sizes (Fig 5). The remaining overlap levels (50%, 25%, and 0%) resulted in the lowest values of these metrics (Fig 5). Finally, sensitivity, specificity, and precision generally did not vary noticeably between window sizes for most activities (Fig 5).

### Chaser models

**Fig 6:**
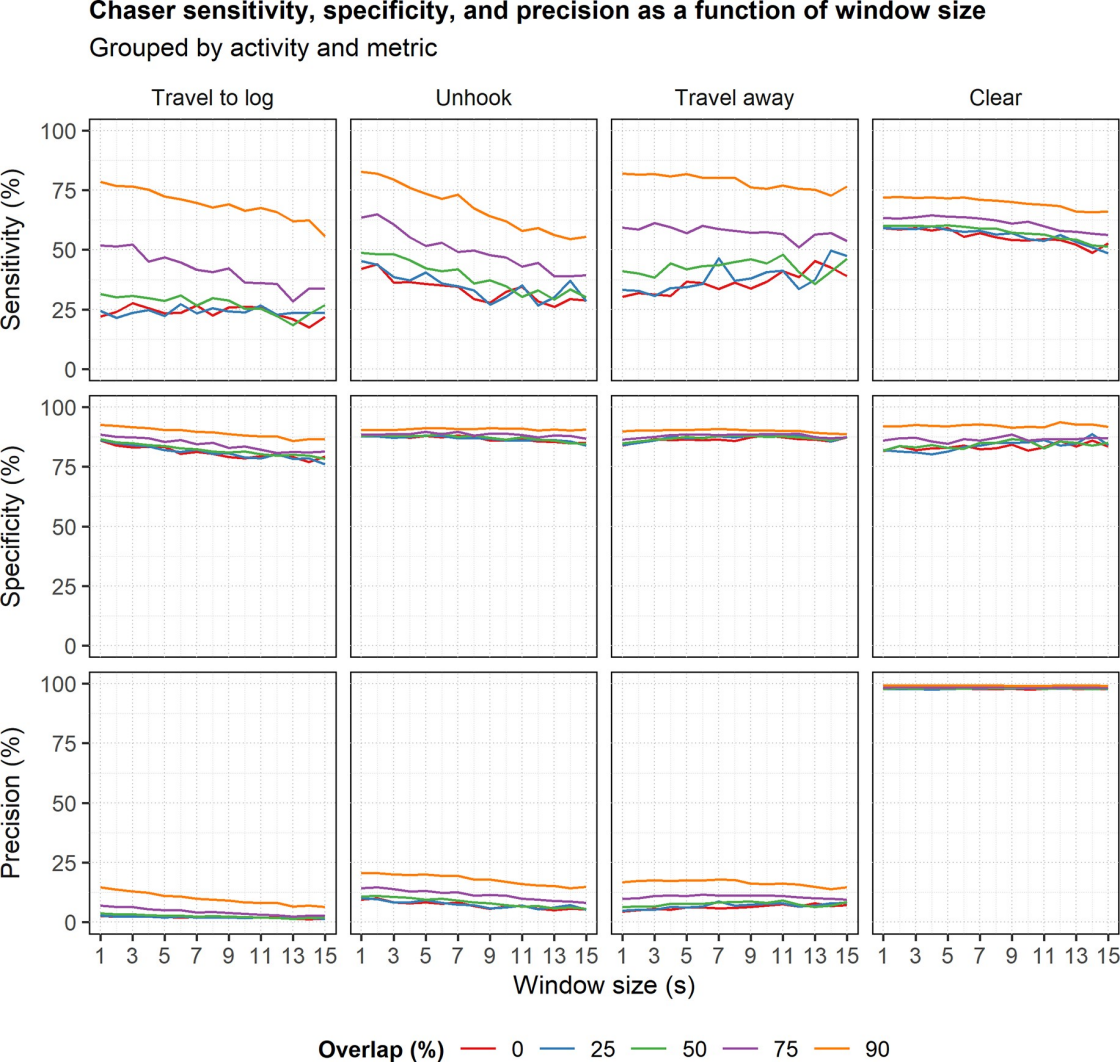
Chaser sensitivity, specificity, and precision as a function of window size. The plots are grouped by metric and work activity. Line color indicates window overlap level.

The sensitivity, specificity, and precision of the chaser models were highest when using sliding windows with 90% overlap for all activities and window sizes (Fig 6). Using sliding windows with 75% overlap resulted in the second-highest values of these same metrics for many activities and window sizes (Fig 6). The remaining overlap levels (50%, 25%, and 0%) generally resulted in the lowest values of these metrics (Fig 6). Additionally, sensitivity tended to decrease as window size increased for most activities (Fig 6, first row) while specificity decreased slightly with increasing window size for the *travel to log* activity but did not show much variation with window size for the other three activities (Fig 6, second row). Precision decreased with increasing window size for the *travel to log* and *unhook* activities at the higher overlap levels but did not vary as noticeably for the *travel away* and *clear* activities (Fig 6, third row).

### Model selection

**Fig 7:**
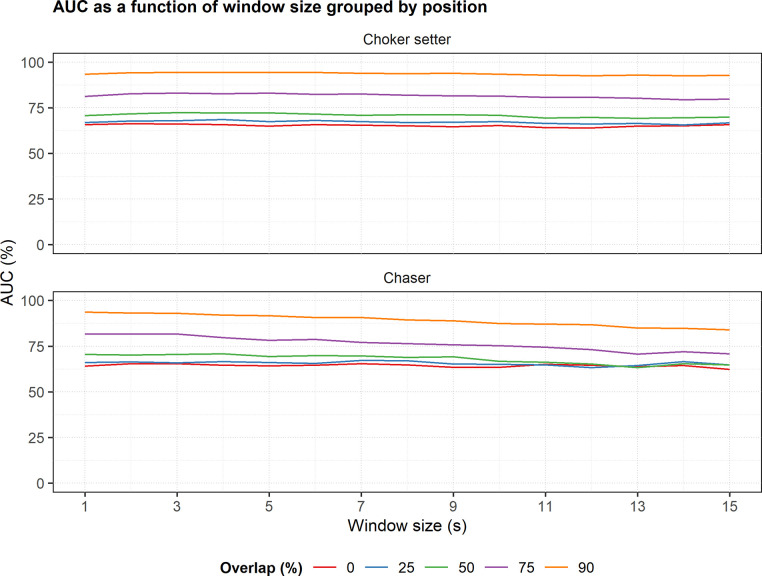
Choker setter and chaser multiclass AUC as a function of window size. The plots are grouped by worker type (choker setter or chaser). Line color indicates window overlap level.

The multiclass AUC plot indicates that the 90% overlap level resulted in the highest AUC values across all window sizes for both the choker setter and chaser models (Fig 7). AUC did not vary much with window size for the choker setter models (Fig 7, top row). For the chaser models, AUC decreased with increasing window size for the 90% and 75% overlap levels but varied less with window size for the other overlap levels (Fig 7, bottom row). A 3-s window with 90% overlap had the highest AUC (94.42%) for the choker setter models (Fig 7, top row) and a 1-s window with 90% overlap had the highest AUC (93.62%) for the chaser models (Fig 7, bottom row). Thus, these two models were chosen as the optimal choker setter and chaser models.

Sensitivity for the selected choker setter model (3-s window, 90% overlap) ranged from 76.95% to 83.59% for the four activities (Table 4). Precision for this model ranged from 41.42% to 97.08% for the four activities, specificity ranged from 91.36% to 94.73%, F_1_ values ranged from 0.55 to 0.86, and balanced accuracy ranged from 85.02% to 88.90% (Table 4). The confusion matrix for the selected choker setter model (3-s window, 90% overlap) illustrates that the *set choker* activity was most often confused with *travel to log* (Table 5). *Travel away* was somewhat equally confused with the other three activities (*set choker*, *travel to log*, and *clear*), while *travel to log* was most often confused with either *set choker* or *clear* (Table 5). Finally, *clear* was most often confused with either *set choker* or *travel to log* (Table 5).

**Table 4:** Accuracy metrics for the best choker setter model (created with a 3-s window and 90% overlap).

Activity	Sensitivity (%)	Precision (%)	Specificity (%)	F_1_	Balanced Accuracy (%)
Travel to log	78.42	51.68	91.63	0.62	85.02
Set choker	83.59	57.82	91.36	0.68	87.48
Travel away	83.06	41.42	94.73	0.55	88.90
Clear	76.95	97.08	93.72	0.86	85.33

**Table 5:** Confusion matrix for the best choker setter model (created with a 3-s window and 90% overlap).

		Actual			
		Set choker	Travel away	Travel to log	Clear
**Predicted**	**Set choker**	18655	455	1898	11254
	**Travel away**	888	6409	554	7622
	**Travel to log**	1743	365	14455	11407
	**Clear**	1031	487	1526	101095

Sensitivity for the selected chaser model (1-s window, 90% overlap) ranged from 71.95% to 82.75% for the four activities (Table 6). Precision for this model ranged from 14.74% to 99.16% for the four activities, specificity ranged from 89.76% to 92.43%, F_1_ values ranged from 0.25 to 0.83, and balanced accuracy ranged from 81.97% to 86.54% (Table 6). The confusion matrix for the selected chaser model (1-s window, 90% overlap) shows that *clear* was most often confused with *travel away* but was also frequently mistaken for *unhook* (Table 7). *Travel away* was most often confused with either *unhook* or *clear*, while *travel to log* was most often mistaken for *clear* (Table 7). Finally, *unhook* was most often confused with either *clear* or *travel away* (Table 7).

**Table 6:** Accuracy metrics for the best chaser model (created with a 1-s window and 90% overlap).

Activity	Sensitivity (%)	Precision (%)	Specificity (%)	F_1_	Balanced Accuracy (%)
Travel to log	78.53	14.74	92.43	0.25	85.48
Unhook	82.75	20.66	90.34	0.33	86.54
Travel away	81.93	16.75	89.76	0.28	85.84
Clear	71.95	99.16	91.99	0.83	81.97

**Table 7:** Confusion matrix for the best chaser model (created with a 1-s window and 90% overlap).

		Actual			
		Clear	Travel away	Travel to log	Unhook
**Predicted**	**Clear**	366814	972	1022	1099
	**Travel away**	53458	11028	372	978
	**Travel to log**	39715	409	7061	714
	**Unhook**	49828	1052	536	13385

## Discussion

Our results show that activity recognition models based on smartwatch accelerometers can characterize work activities for rigging crew workers setting and disconnecting log chokers on cable logging operations, with the best model sensitivities ranging from 76.95% to 83.59% for choker setters and from 71.95% to 82.75% for chasers. While not all activities met our objective of 80% sensitivity, these values are consistent with models based on smartwatches and wrist-worn accelerometers developed for other activities in previous studies [27, 29, 32, 33, 35, 54–56]. The benchmark of 80% sensitivity was established ahead of time as part of project development. While we recognize that a combination of metrics may be more suitable for future use, particularly when dealing with imbalanced datasets, this was a pilot study intended to help establish methods prior to a larger modeling effort.

In contrast to the fairly high sensitivity values, precision was poor for most activities for both the choker setter and chaser models. This may be due to the imbalanced nature of the data. The proportions of choker setter activities were 10.26% *travel to log*, 12.41% *set choker*, 4.29% *travel away*, and 73.04% *clear*. The proportions of chaser activities were 1.64% *travel to log*, 2.95% *unhook*, 2.45% *travel away*, and 92.95% *clear*. Furthermore, the high CV values in Table 2 suggest there was high variability within individual work cycle elements, especially within the *clear* activity for both the choker setters (CV = 235.65%) and chasers (CV = 182.06%), which likely resulted at least in part from the inclusion of delay time in work elements. Additional variability may have been introduced to the *clear* activity due to the fact that *clear* encompassed everything workers did outside of the other three work elements. The combination of this variability with the large proportion of time workers spent in the clear likely reduced prediction accuracy and could account for the generally low precision and F_1_ values. Since this form of analysis is very new in natural resources work, there is relatively little prior research on how delay, misclassified activities, or low precision in general may impact real-time summaries of work, or worker health and safety considerations, when interpreted in practice. Rather than a shortcoming of our study, we feel this is an important consideration for subsequent research and for development of wearable-based digital health and safety analytics for operational forestry. Additionally, conventional time study analysis distinguishes between quantifying individual productive cycle elements and quantifying overall productive and delay components of occupational work. Future modeling studies may benefit from distinguishing sampling efforts to quantify these separately. This may influence the way in which low precision and high false positive rates affect metrics such as productivity that may be calculated from model predictions in real-time. Thus, future work should consider how *clear* is defined and whether delay times are included in sampling and developed models in an effort to improve precision. Future work should also address issues of low precision through improved data collection, processing, and modeling (e.g., utilizing video recording, faster sampling rates, different sensors, deep learning, additional features, etc.), all of which are discussed below.

The best model performance metrics for both workers were obtained using the highest level of window overlap (90%) and smaller window sizes. The increasing accuracy we obtained with higher levels of window overlap is consistent with previous work [45]. However, many wrist-based models developed with either no overlap [27, 29, 56] or 50% overlap [54, 55] have achieved high accuracies. In terms of window size, previous work has suggested that simpler, more repetitive activities may be accurately captured with shorter windows, while more complex, less repetitive activities may need longer windows [34, 55]. In general, we observed slightly decreasing performance metrics with increasing window size. While some previous studies have found improved accuracies with increasing window size [34, 56], others have observed that increasing window size did not result in significant model improvement [55] and have developed accurate wrist-based models using smaller window sizes [35, 54, 55].

Smartwatches, smartphones, and other wearables generally have limited resources in terms of battery power, memory, storage, and computational power, so the effects of window size, window overlap, and sampling rate must be considered when designing and implementing occupational activity recognition models [31, 42, 111, 112]. For instance, using longer windows [45, 111], higher levels of window overlap [45], and faster sampling rates [31, 42] requires more computational resources during implementation. In our study, the difference between the 90% and 75% overlap levels was noticeable for many of the model accuracy metrics. While lower levels of overlap may be preferable for real-time implementation on resource-limited devices such as smartwatches, the improved accuracies observed with the higher overlap levels seem to justify the additional computational power needed. On the other hand, our results suggest that the highest accuracies for both the choker setter and chaser models can be obtained with smaller window sizes, which indicates real-time implementation may be achieved with relatively minimal computational complexity and fast detection rates. Finally, future work should evaluate the effects of different sampling rates. While previous work has shown that higher frequencies can lead to improved accuracies [42, 111], others have achieved high performance using lower sampling rates such as 2-Hz [111] and 10-Hz [30]. The RawLogger application used to record the watch sensor data in this study did not allow us to adjust the sampling frequency, and Garmin Connect allows a maximum rate of 25-Hz for the accelerometer. However, different devices could be used in future work to assess whether higher frequencies may improve performance and whether lower sampling frequency may achieve similar performance, as this would have the benefit of reducing power consumption on the device.

Other considerations related to performance and resource consumption on wearable devices are the types of features used for classification and the tradeoffs between classical machine learning and deep learning methods. We chose to use time domain features primarily because they are less computationally complex and consume less energy during implementation [42, 113]. Additionally, it has been shown that the use of frequency domain features should be paired with faster sampling rates in order to achieve high model performance [42]. However, the use of frequency domain features should be investigated as a potential method of improving model performance [114]. Similarly, we chose to use a traditional machine learning approach because deep learning methods are generally more computationally expensive [47]. As devices become more powerful, deep learning offers opportunities to overcome some of the limitations of classical machine learning [48–50].

Activity recognition models based on wearables such as smartphones and smartwatches have practical applications for quantifying productivity and reporting work analytics to support digital health and safety. In terms of rigging crew productivity, models may inform the real-time reporting of productive work elements such as mean time per turn, number of chokers set or disconnected per turn, and related analytics. Models developed using conventional time studies in forestry have been limited to very specific operations and time periods under consideration, or deployed broadly in generalized predictive models (e.g., Bell et al. [115]). Utilizing our activity recognition models and subsequent, improved models using similar approaches makes it possible to quantify work day-in, day-out over the course of the work week and indefinitely in the future when incorporated into predictive apps on smartwatches. This is a major advancement and opens new possibilities for analysis of big data accumulated over time. For example, when summarized at the individual and group level, this information could be used to improve productivity, reduce costs, and enhance work quality and worker safety by allowing workers to adapt treatment methods in near real-time. Furthermore, in broader meta-analysis, use of wearables to quantify productivity with greater temporal resolution than traditional time study techniques may provide opportunities for improving estimates of work productivity and treatment costs across various stand and site conditions and spatial scales. To better quantify worker health and safety, activity recognition model predictions may be paired simultaneously with fall detection algorithms to inform smart alerts indicating lack of movement associated with potential incidents. These alerts may then be sent to coworkers at the jobsite using emerging technologies designed to facilitate communication and data-sharing to improve safety in remote work areas. Model predictions may also be combined with other health-related data, such as heart rate, heat stress, and sleep metrics, to develop alerts that would be triggered when demanding work activities occur in conjunction with high physical exertion levels, increased heat stress, excess workload, or a combination of these factors. Finally, pairing activity recognition model predictions with real-time location information may be used to inform safety alerts related to proximity to coworkers, jobsite hazards such as snags or falling trees, and heavy equipment. This information could also be used in post-hoc analyses to better characterize the activities and other location- and health-related factors preceding accidents and near miss events.

One limitation to our study was reduced visibility when observing workers. Choker setters commonly work on steep hillsides, often among shrubs and other vegetation. At times, this made it difficult to visually observe start and stop times for component work cycle elements with precision. Chasers working at the landing were occasionally difficult to see when the yarder’s movement shielded the chaser from view. A related limitation was a slight difference between the internal clocks of the smartwatches and the phones used to record the start and stop times for each activity. While these differences were small, there was no practical way to correct them in the field as they occasionally shift within a 24-hour period. This introduced additional error when assigning labels to the watch sensor data and is an important consideration in future remote occupational digital health applications, particularly for mobile and wearable devices that sync National Institute of Standards and Technology (NIST) time via internet connectivity.

Another limitation to the study was the short duration of many of the work activity cycles, primarily for the chaser. The chaser often runs in to and away from the yarded logs (both of which may last only a few seconds), and unhooking can be very quick (i.e., 3–5 s). This makes it difficult to visually detect and record accurate start and stop times for the rapid cycle elements that result. While this may explain why the shorter window lengths generally resulted in higher accuracies, it also made the activities more difficult to capture and model. Development of subsequent wearable- and mobile-based activity recognition for occupational safety may benefit from use of video concurrently with smartwatch sensor data collection rather than direct visual observation, in order to improve work element detection and support model training. For example, use of body camera videography coupled with post-hoc analysis to quantify work elements for low visibility tasks in forestry may improve model development.

A few areas that should be considered in future research include evaluating additional sensors, utilizing devices on different body parts, and incorporating more mechanistic approaches to modeling. The Garmin watches used in this study were only capable of recording raw accelerometer data. However, the incorporation of gyroscope sensor measurements into model development in future studies could potentially help to strengthen predictive power. While previous research has indicated that watch accelerometers perform better than watch gyroscopes for activity recognition [29], others have found that using both accelerometers and gyroscopes increases accuracy for smartphone-based models [52] and watch-based models [32]. Additionally, previous studies have shown that hip-mounted accelerometers may recognize activities like running better than wrist-mounted accelerometers [27] and that using sensors in multiple positions, such as the wrist and pocket, can improve model performance [34]. Thus, future work should evaluate how placing devices on different body parts affects model predictions. For instance, it is possible that using sensors on the torso or feet may more accurately recognize the movements of choker setters and chasers as they travel to and from logs, while wrist-based sensors may be better suited to detecting activities that involve hand motions, like setting and disconnecting chokers. Another consideration when evaluating device locations is that previous research has shown that participants may prefer wrist-worn devices compared to hip-worn devices [116], which has implications for designing a relatively unobtrusive system for real-world adoption. Lastly, use of the random forest machine learning algorithm to model the occupational activities of rigging crew workers is highly empirical and doesn’t necessarily help to foster understanding of the underlying processes affecting work productivity and safety. In future studies, a more mechanistic approach to modeling work movements may better lend itself to identifying causal relationships associated with safety incidents and possible interventions.

Because logging operations are highly variable and our sampling was observational, the amount of data collected from each participant varied. We chose to randomly separate the entire dataset into 2/3 training and 1/3 testing since utilizing either leave-one-subject-out or k-fold cross-validation implemented at the participant level would have meant creating models with varying quantities of data in each iteration. Thus, because our validation methods may randomly include data from participants in both training and testing, the quality of predictive models presented may be overly optimistic. Future research developing similar models may benefit from a different approach that avoids cross-over of participant data in training and testing subsets. Additionally, traditional time and motion and actigraphy analysis in forestry work has generally been based on relatively small studies. Because use of IMU sensors to quantify work in real-time is relatively new, it is unknown whether the mean and variability of data in our study would be fully representative of the broad range of field sites, forest stand conditions, equipment, and weather impacts that affect worker movements in the profession overall. While we believe the quantity of data from each participant used to train and test the models was sufficient, future studies may benefit from collecting a more balanced sample of data from a wider variety of participants. Our goals in this study were to evaluate, at a broad level, the potential for wearable devices to model real-time occupational rigging crew work activities and to provide an example of the methods, modeling approaches, and sampling considerations that are important for developing libraries of generalized forestry work activity recognition models. Prior to use in occupational settings, predictions from real-time models developed, regardless of the statistical validation methodology used in model fitting and analysis, should be further evaluated using data collected independently as part of different field operations reflecting variability in site conditions, weather, workers, and other factors.

Ultimately, future work should include coding the best models developed in this study into a smartwatch application to support real-time characterization of work activities and further validate model predictions in a variety of conditions. Pairing of fall detection with activity recognition model predictions may help to inform development of improved smart alerts to coworkers notifying them of potential jobsite incidents, particularly when paired with real-time GNSS mapping in remote forestry work environments. To advance digital health and safety more broadly, the data resulting from our predictive models, as well as from models developed subsequently for other common forestry work tasks, may be used to quantify day-to-day occupational forestry job functions in high resolution. The resulting work effort data provide a fundamental mechanism through which it may be possible to better quantify factors associated with incident occurrence across forest stand, site, weather, air quality, and other work conditions, particularly when paired with readily available, wearable-based personal health metrics such as sleep activity, heart rate, and heat stress.
